# Endoluminal single-clip traction-assisted endoscopic submucosal dissection: a green tip to reduce waste and cost

**DOI:** 10.1055/a-2462-1649

**Published:** 2024-11-26

**Authors:** Zeyu Wu, Yonggang Ding, Qide Zhang

**Affiliations:** 1 688090 Digestive Endoscopy Center, Affiliated Hospital of Nanjing University of Chinese Medicine, Nanjing, China

A 66-year-old man was referred to our hospital for endoscopic submucosal dissection (ESD) of a descending colonic laterally spreading tumor with pit pattern type IIIL and IV _V_ ( Fig. 1 ). The lesion was located on the lower side with respect to gravity when the patient underwent endoscopic operation in the left lateral position. We designed an endoluminal single-clip traction (ECT) method to assist ESD.

**Fig. 1:**
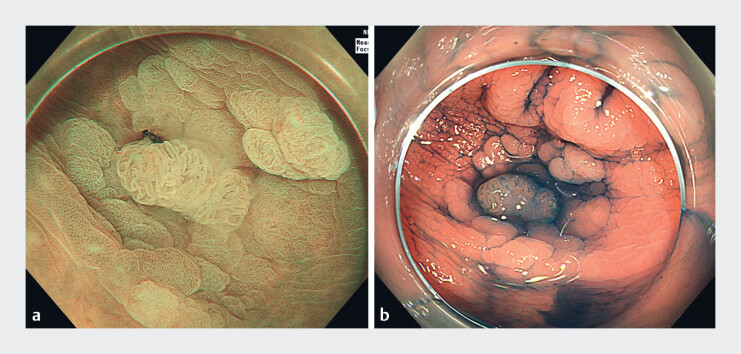
A laterally spreading tumor in the descending colon on the lower side with respect to gravity, with pit pattern type IIIL and IV _V_ .

After completing circumferential mucosal incision, we used a reopenable SureClip (Micro-Tech, Nanjing, China) to clamp the mucosal flap to the opposite mucosal wall and simultaneously applied gas suction. Once this was achieved, gradual adjustment of gas volume (gas +~+++) was utilized to maintain traction tension for further dissection ( Fig. 2 ). Once the lesion had been dissected ( Fig. 3 ), a snare was used to grasp the foot of the clip and retrieve the specimen ( Fig. 4 ). The whole process is shown in Video 1 .

**Fig. 2:**
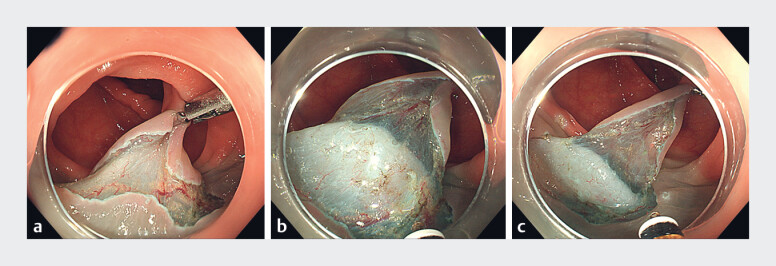
Endoluminal single-clip traction-assisted colonic endoscopic submucosal dissection to maintain the traction effect sufficiently (gas+~+++).

**Fig. 3:**
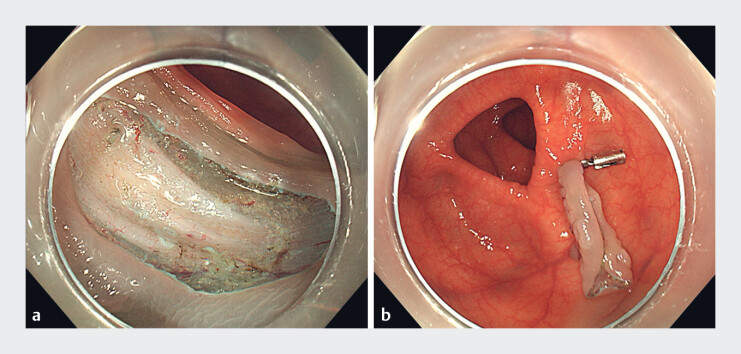
The wound after endoscopic submucosal dissection and the resection specimen attached to the colonic wall by a clip.

**Fig. 4:**
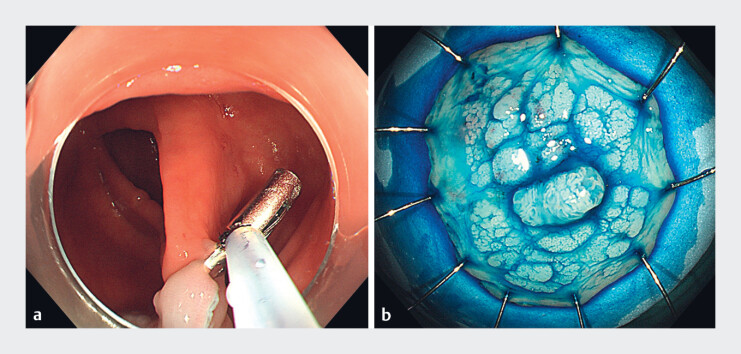
A snare was used to grasp the foot of the clip and retrieve the specimen.

Endoluminal single-clip traction assisted endoscopic submucosal dissection for a colonic laterally spreading tumor.Video 1

The patient was discharged uneventfully on Day 3, and the pathology revealed superficially serrated adenoma ( Fig. 5 ).

**Fig. 5:**
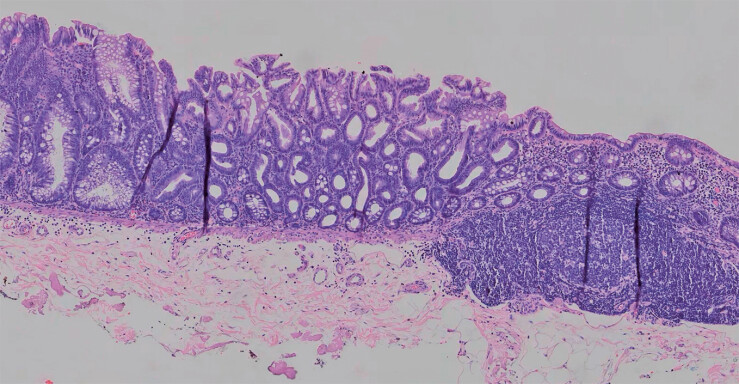
The pathology of the lesion. Hematoxylin and eosin stain (×100).

ESD remains a challenge for endoscopists because of anatomical structure, such as the thin colorectal wall or curved intestinal cavity. It is of vital importance to maintain a clear visual field during dissection and to maintain a stable dissection plane. Therefore, many traction methods are utilized to assist colorectal ESD, such as double-clip and rubber band traction 1 , and S-O clip (Zeon Medical, Tokyo, Japan) traction 2 . One important step before starting ESD is to note the direction of gravity. Fluid collection should occur on the opposite side of the lesion to avoid pooling in the resection area, and traction by gravity would provide a good visual field. In order to dissect the submucosal tissue smoothly, it is necessary to utilize gravity and change the patient’s position for traction. Nevertheless, we utilized only one reopenable clip to conduct ECT-assisted ESD without having to consider the direction of gravity; the waste and cost of traction was therefore kept to a minimum.

Endoscopy_UCTN_Code_TTT_1AQ_2AD_3AD

## Notes

Endoscopy E-Videos https://eref.thieme.de/e-videos
*E-Videos* is an open access online section of the journal *Endoscopy* , reporting on interesting cases and new techniques in gastroenterological endoscopy. All papers include a high-quality video and are published with a Creative Commons CC-BY license. Endoscopy E-Videos qualify for HINARI discounts and waivers and eligibility is automatically checked during the submission process. We grant 100% waivers to articles whose corresponding authors are based in Group A countries and 50% waivers to those who are based in Group B countries as classified by Research4Life (see: https://www.research4life.org/access/eligibility/ ). This section has its own submission website at https://mc.manuscriptcentral.com/e-videos .
